# Supplementary material for: Regularized selection indices for breeding value prediction using hyper-spectral image data
Source: Sci Rep. 2020 May 18;10:8195. doi: 10.1038/s41598-020-65011-2 (PMC7235263; doi:10.1038/s41598-020-65011-2)
Supplement: Supplementary file 1 — Supplementary information. [file 41598_2020_65011_MOESM1_ESM.pdf]

Supplementary information

Regularized selection indices for breeding value prediction using hyper-spectral image data

Marco Lopez-Cruz[1], Eric Olson[1], Gabriel Rovere[2,3,4], Jose Crossa[6], Susanne Dreisigacker[6], Suchismita Mondal[6], Ravi Singh[6], and Gustavo de los Campos[3,4,5,*]

[1]Department of Plant, Soil and Microbial Sciences, Michigan State University, USA

[2]Department of Animal Science, Michigan State University, USA

[3]Department of Epidemiology and Biostatistics, Michigan State University, USA

[4]Institute for Quantitative Health Science and Engineering, Michigan State University, USA

[5]Department of Statistics and Probability, Michigan State University, USA

[6]International Maize and Wheat Improvement Center (CIMMYT), Mexico

*Correspondence and request should be addressed to G.D.L.C. (e-mail: gustavoc@msu.edu).

This file contains:

- Supplementary Figs. S1-S8
- Supplementary Table S1

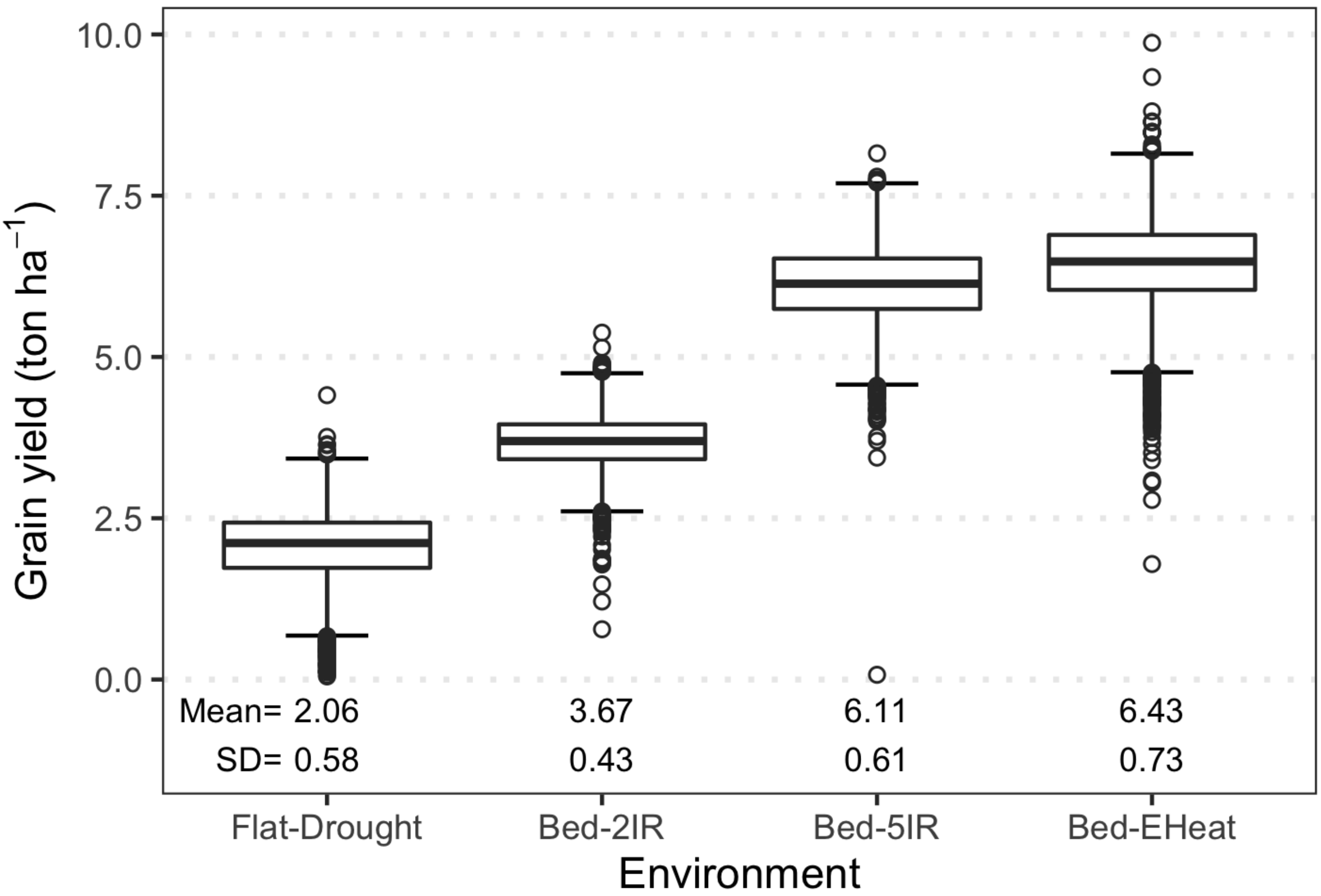

Supplementary Fig. S1. Box-plot of grain yield phenotypic records by environmental condition. $n \approx 3200$ observations within environment. SD: standard deviation.

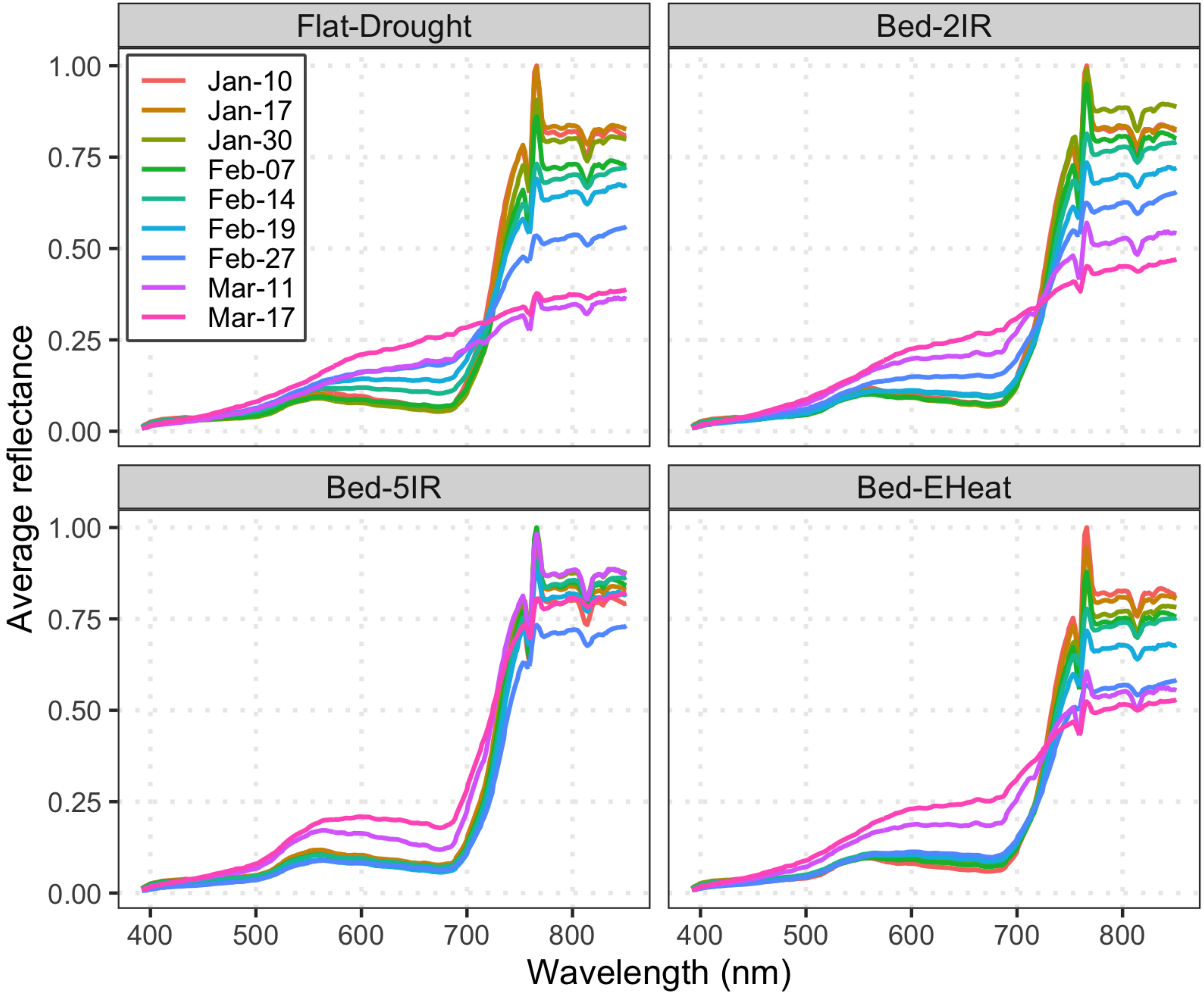

Supplementary Fig. S2. Light reflectance patterns as function of the wavelength. Each line represents the mean (across $n \approx 3200$ observations) reflectance for each waveband, within time-point (flight date). Within each environment, means were scaled to lie within 0 and 1 by dividing them by the maximum average.

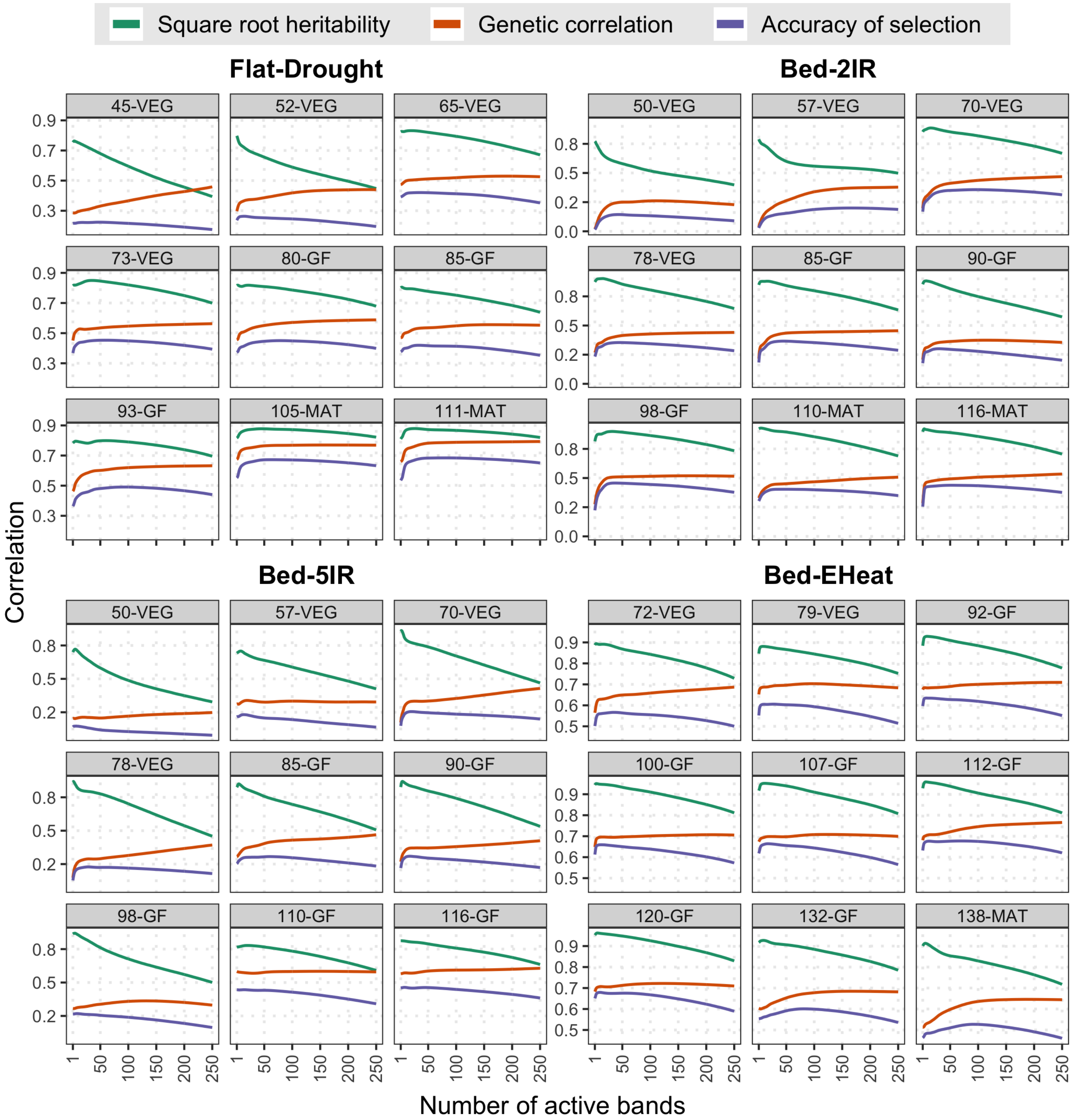

Supplementary Fig. S3. Accuracy of indirect selection of L1-PSI and its components. Square root heritability, genetic correlation and accuracy of indirect selection, all averaged over 100 training-testing partitions versus the number of bands entering in the index; by time-point (DAS=days after sowing, Stage: VEG=vegetative, GF=grain filling, or MAT=maturity) within environment.

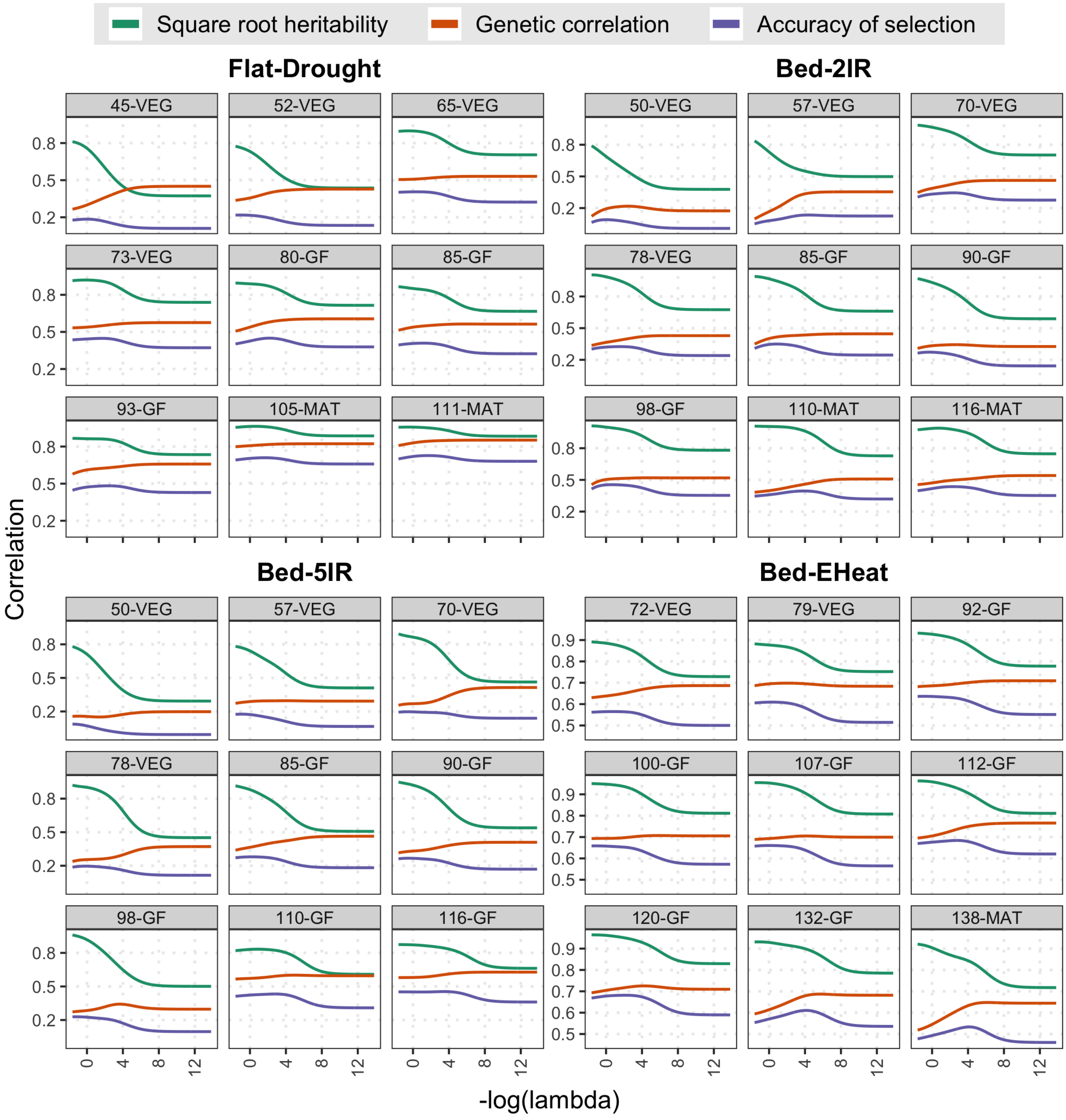

Supplementary Fig. S4. Accuracy of indirect selection of L2-PSI and its components. Square root heritability, genetic correlation and accuracy of indirect selection, all averaged over 100 training-testing partitions versus the penalization parameter (λ, logarithm scale) used to build the index; by time-point (DAS=days after sowing, Stage: VEG=vegetative, GF=grain filling, or MAT=maturity) within environment.

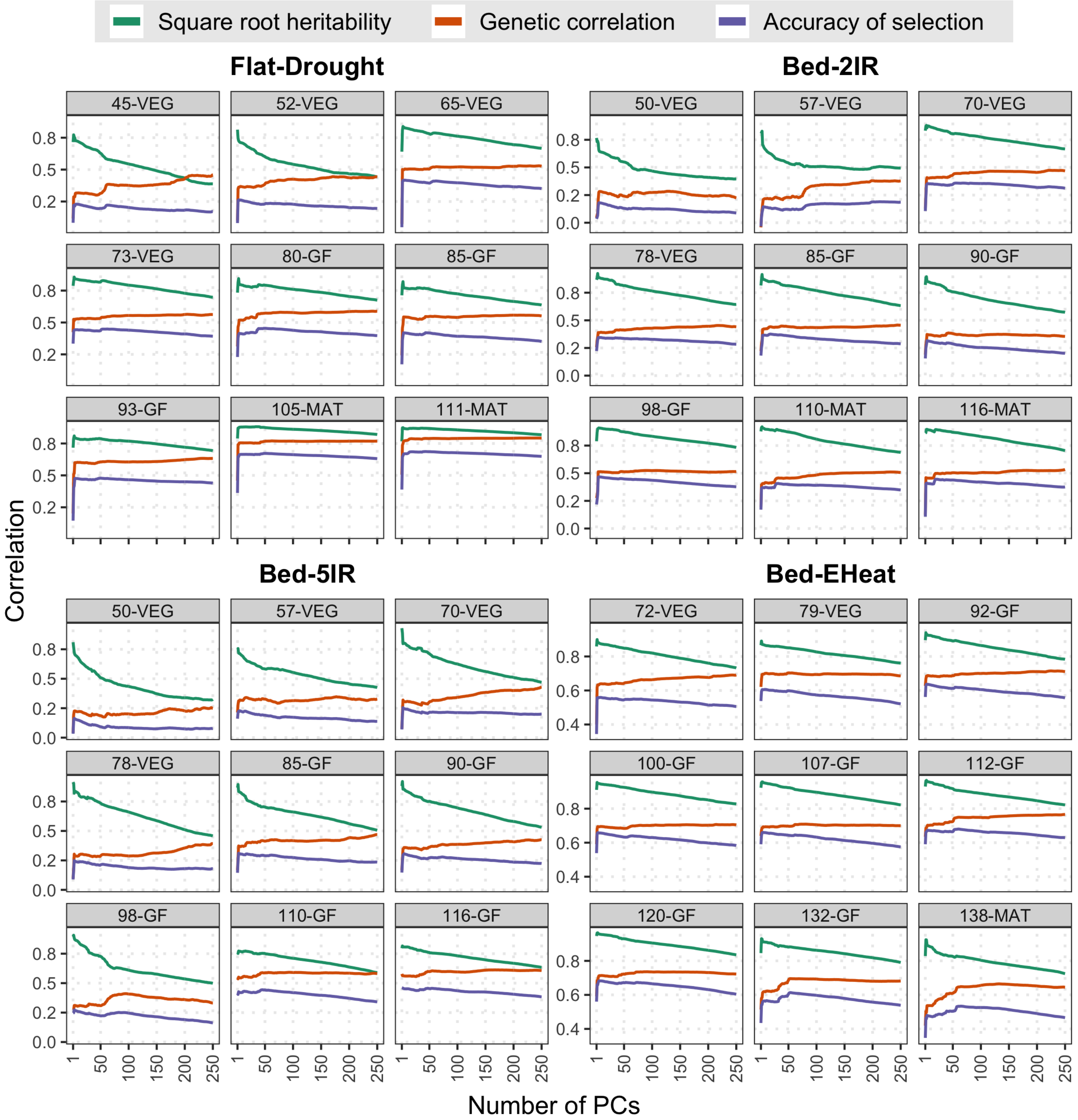

Supplementary Fig. S5. Accuracy of indirect selection of PC-SI and its components. Square root heritability, genetic correlation and accuracy of indirect selection, all averaged over 100 training-testing partitions versus the number of principal components used to build the index; by time-point (DAS=days after sowing, Stage: VEG=vegetative, GF=grain filling, or MAT=maturity) within environment.

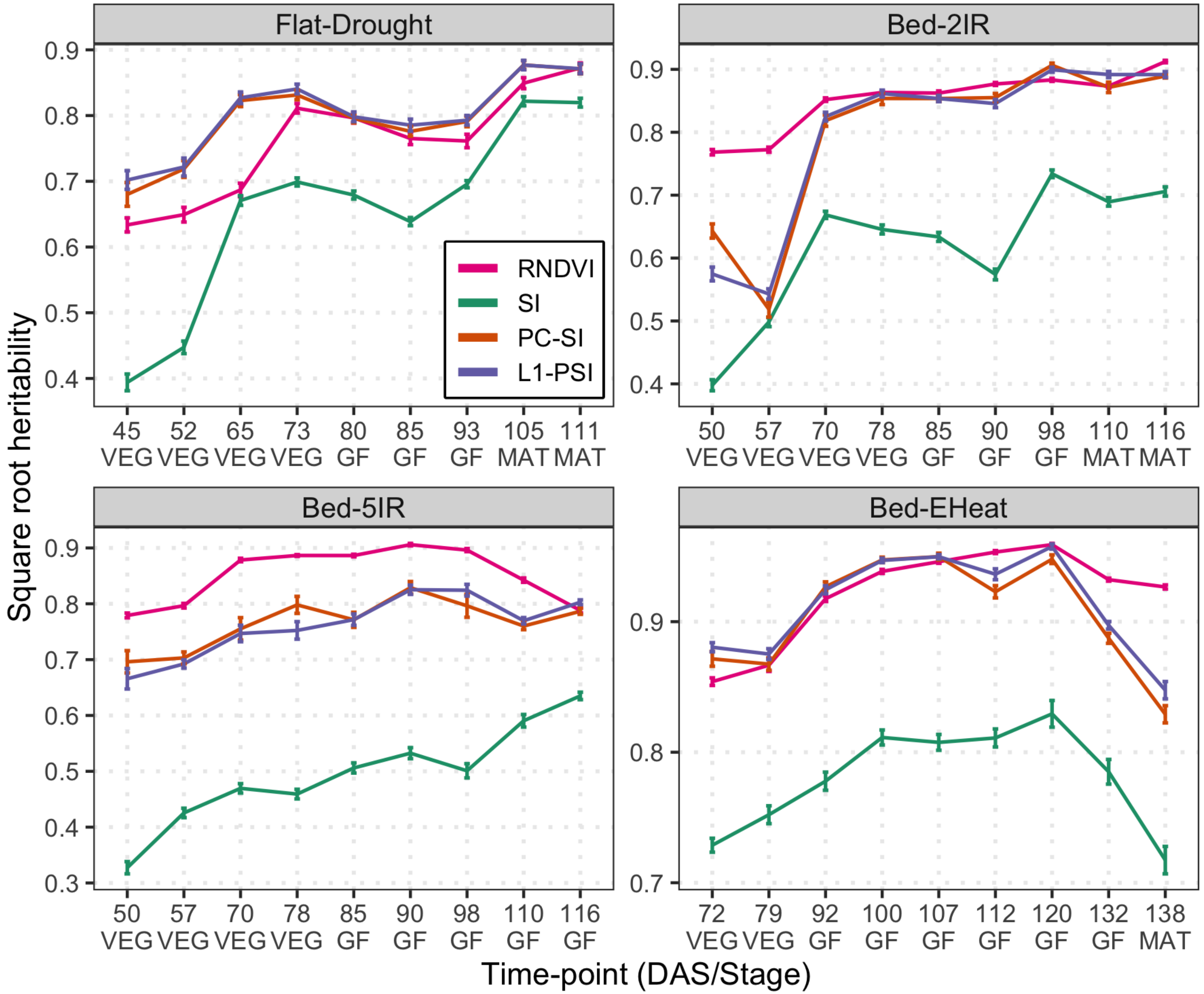

Supplementary Fig. S6. Square root of heritability of the standard (SI), of the regularized (PC-SI and L1-PSI) selection indices, and of the RNDVI. The lines provide the average square root heritability over 100 training-testing partitions. Vertical lines represent a 95% CI for the average. The horizontal axis give the time-point at which images were collected and are expressed in both days after sowing (DAS) and stages (VEG=vegetative, GF=grain filling, MAT=maturity).

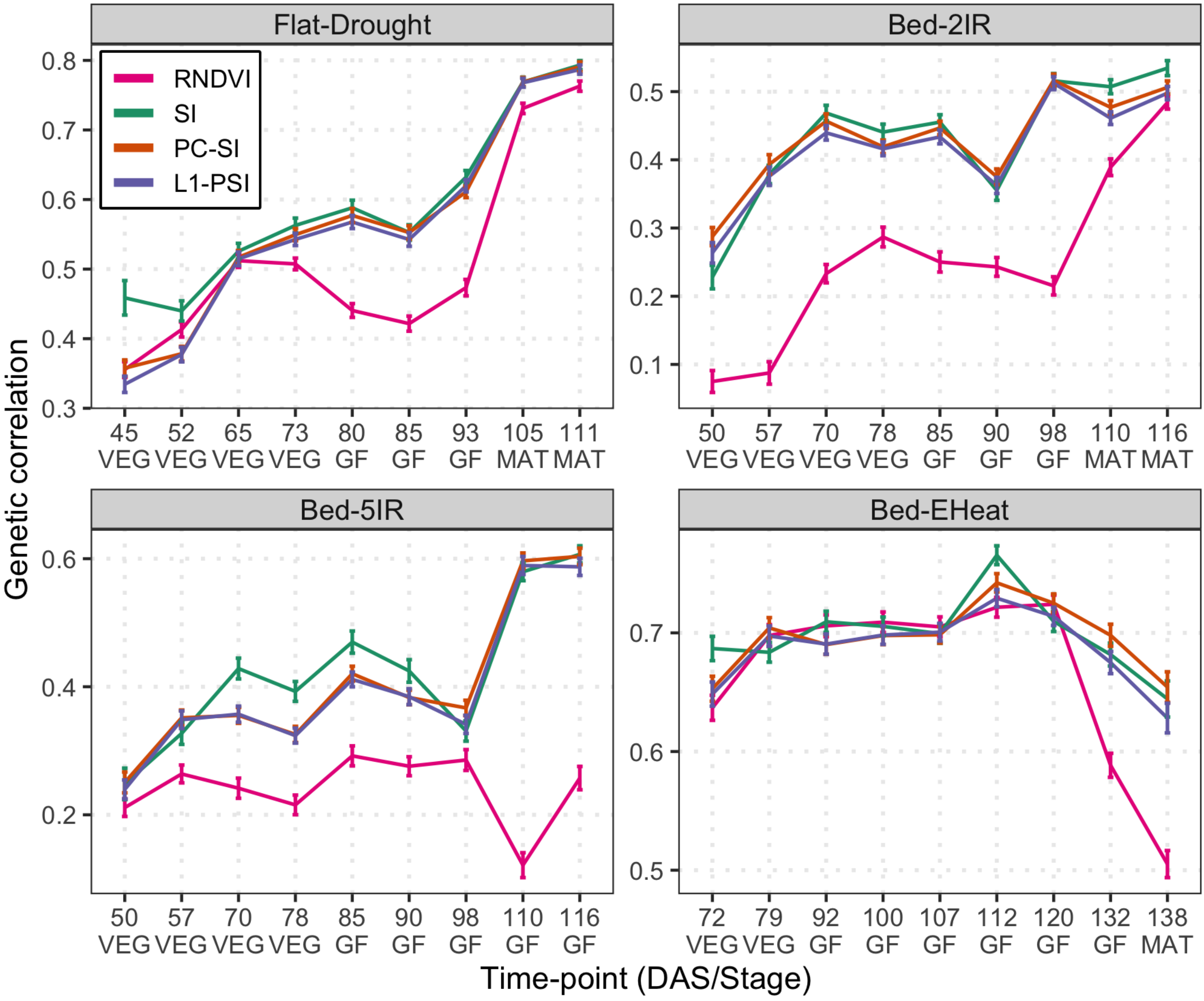

Supplementary Fig. S7. Genetic correlation between grain yield and all: the standard (SI), the regularized (PC-SI and L1-PSI) selection indices, and the RNDVI. The lines provide the average genetic correlation over 100 training-testing partitions. Vertical lines represent a 95% CI for the average. The horizontal axis give the time-point at which images were collected and are expressed in both days after sowing (DAS) and stages (VEG=vegetative, GF=grain filling, MAT=maturity).

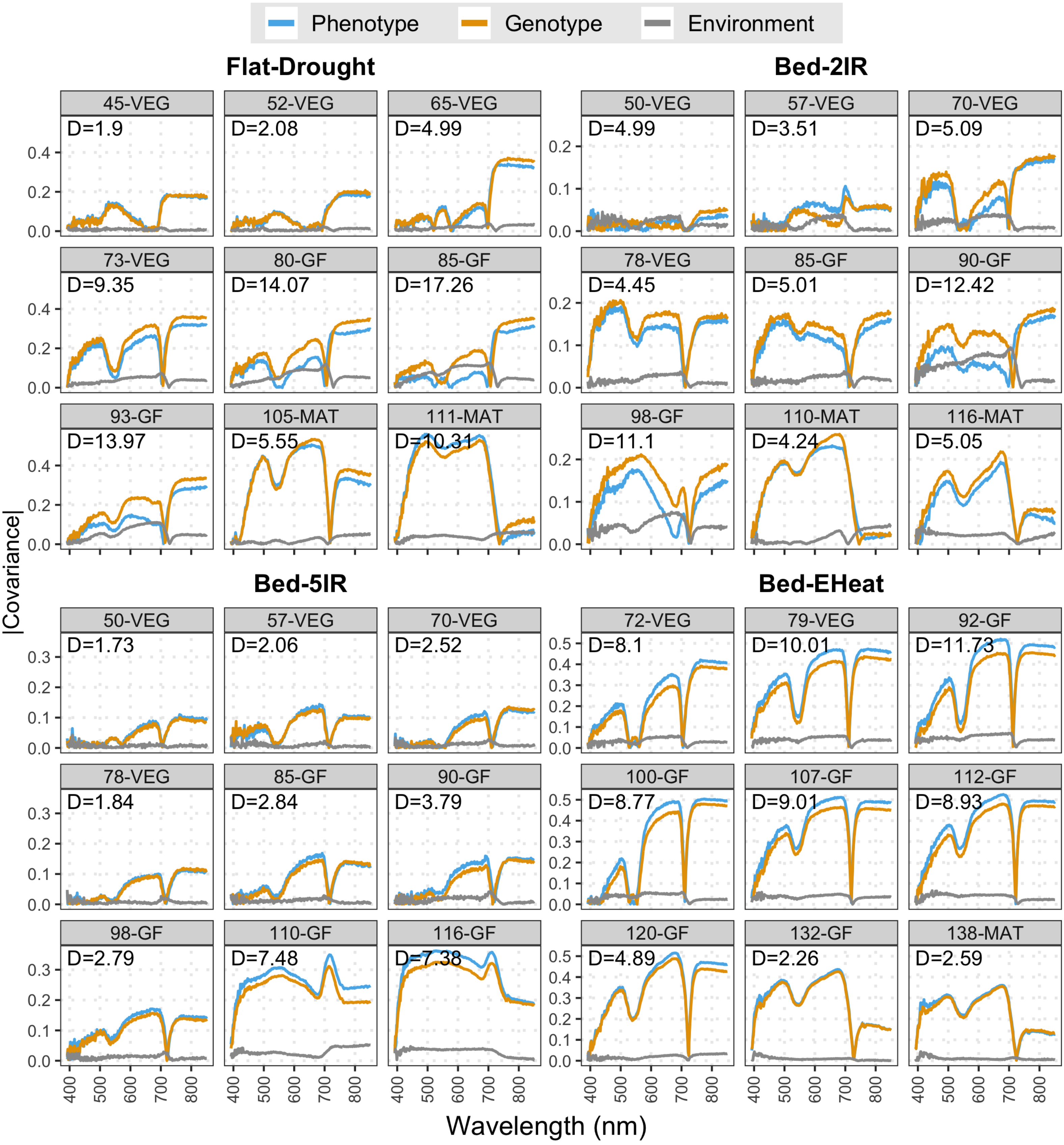

Supplementary Fig. S8. Phenotypic, genetic, and environmental covariances (absolute value) between wavebands and grain yield. 'D': discrepancy between phenotypic and genetic covariances as measured by the sum of the absolute differences; by time-point (DAS: days after sowing, Stage: VEG=vegetative, GF=grain filling, MAT=maturity) within environment.

Env/time-point		Phenotypic prediction				Genotypic prediction			
		PCR	L1-Phen	RNDVI	GNDVI	SI	PC-SI	L1-PSI	L2-PSI
Flat-Drought	45-VEG	0.24 a	0.23 a	0.23 a	0.21 b	0.18 c	0.24 a	0.23 a	0.24 a
	52-VEG	0.27 ab	0.27 ab	0.27 ab	0.25 b	0.20 c	0.27 a	0.27 a	0.27 ab
	65-VEG	0.42 a	0.42 a	0.35 b	0.35 b	0.35 b	0.43 a	0.43 a	0.42 a
	73-VEG	0.45 ab	0.45 ab	0.41 cd	0.43 bc	0.39 d	0.46 a	0.46 a	0.46 a
	80-GF	0.44 bc	0.43 c	0.35 e	0.39 d	0.40 d	0.46 a	0.45 ab	0.46 a
	85-GF	0.41 abc	0.40 cd	0.32 f	0.39 d	0.35 e	0.43 a	0.43 ab	0.43 a
	93-GF	0.46 bc	0.47 abc	0.36 e	0.45 cd	0.44 d	0.48 ab	0.49 a	0.49 a
	105-MAT	0.67 a	0.67 a	0.62 b	0.64 b	0.63 b	0.68 a	0.67 a	0.68 a
	111-MAT	0.68 ab	0.68 ab	0.67 bc	0.64 d	0.65 cd	0.69 ab	0.69 ab	0.69 a
	Multi	0.68 cd	0.68 bcd	0.68 d	0.65 e	0.00 f	0.70 ab	0.70 abc	0.70 a
Bed-2IR	50-VEG	0.18 a	0.14 cd	0.00 f	0.12 d	0.09 e	0.18 a	0.15 bc	0.16 ab
	57-VEG	0.19 a	0.19 a	0.00 c	0.03 b	0.19 a	0.20 a	0.20 a	0.20 a
	70-VEG	0.37 a	0.36 a	0.20 c	0.21 c	0.31 b	0.37 a	0.36 a	0.38 a
	78-VEG	0.35 a	0.35 a	0.25 c	0.30 b	0.28 b	0.36 a	0.36 a	0.36 a
	85-GF	0.37 a	0.36 a	0.22 c	0.30 b	0.29 b	0.38 a	0.37 a	0.38 a
	90-GF	0.30 abcd	0.29 cd	0.21 e	0.28 d	0.20 e	0.32 a	0.31 abc	0.32 ab
	98-GF	0.45 a	0.46 a	0.18 d	0.35 c	0.38 b	0.47 a	0.46 a	0.46 a
	110-MAT	0.40 abc	0.39 bc	0.34 d	0.39 c	0.35 d	0.42 a	0.41 ab	0.42 a
	116-MAT	0.44 a	0.44 a	0.44 a	0.39 b	0.38 b	0.45 a	0.44 a	0.45 a
	Multi	0.53 cd	0.53 d	0.46 e	0.40 f	0.01 g	0.55 ab	0.54 bc	0.56 a
Bed-5IR	50-VEG	0.18 a	0.17 ab	0.16 ab	0.15 b	0.08 c	0.17 ab	0.16 ab	0.16 ab
	57-VEG	0.25 a	0.25 a	0.21 c	0.21 bc	0.14 d	0.25 a	0.24 a	0.24 ab
	70-VEG	0.27 a	0.26 a	0.21 b	0.19 b	0.20 b	0.27 a	0.27 a	0.26 a
	78-VEG	0.26 a	0.24 a	0.19 b	0.19 b	0.18 b	0.26 a	0.24 a	0.26 a
	85-GF	0.32 a	0.32 a	0.26 b	0.25 b	0.24 b	0.32 a	0.32 a	0.33 a
	90-GF	0.31 a	0.31 a	0.25 c	0.28 b	0.22 d	0.32 a	0.32 a	0.32 a
	98-GF	0.30 a	0.29 a	0.26 b	0.25 b	0.16 c	0.29 a	0.28 a	0.28 a
	110-GF	0.46 a	0.45 a	0.10 d	0.22 c	0.34 b	0.45 a	0.45 a	0.45 a
	116-GF	0.47 a	0.47 a	0.20 d	0.34 c	0.38 b	0.47 a	0.47 a	0.47 a
	Multi	0.54 a	0.54 a	0.32 c	0.37 b	0.00 d	0.54 a	0.55 a	0.55 a
Bed-EHeat	72-VEG	0.57 a	0.57 a	0.54 b	0.53 b	0.50 c	0.57 a	0.57 a	0.57 a
	79-VEG	0.61 a	0.61 a	0.60 a	0.58 b	0.51 c	0.61 a	0.61 a	0.61 a
	92-GF	0.64 a	0.64 a	0.65 a	0.63 a	0.55 b	0.64 a	0.64 a	0.64 a
	100-GF	0.66 a	0.66 a	0.67 a	0.65 a	0.57 b	0.66 a	0.66 a	0.66 a
	107-GF	0.66 a	0.66 a	0.67 a	0.66 a	0.56 b	0.66 a	0.67 a	0.66 a
	112-GF	0.68 ab	0.68 ab	0.69 a	0.66 b	0.62 c	0.68 a	0.68 a	0.69 a
	120-GF	0.69 a	0.68 a	0.69 a	0.66 b	0.59 c	0.69 a	0.68 a	0.68 a
	132-GF	0.62 a	0.61 a	0.55 b	0.54 b	0.54 b	0.62 a	0.61 a	0.61 a
	138-MAT	0.54 a	0.53 a	0.47 b	0.46 b	0.46 b	0.54 a	0.53 a	0.54 a
	Multi	0.71 a	0.70 a	0.70 a	0.67 b	0.00 c	0.71 a	0.71 a	0.72 a

Supplementary Table S1. Accuracy of indirect selection (average over 100 training-testing partitions) for best phenotypic prediction (principal components (PCR), L1-penalized prediction (L1-Phen), RNDVI, and GNDVI) and for best genotypic prediction (standard SI, optimal PC-SI, L1-PSI, and L2-PSI). Each row contains results for each environment and time-point (DAS: days after sowing, Stage: VEG= vegetative, GF=grain filling, MAT=maturity). Models with the same letter (within each row) are not significantly different from each other (α=0.05, ANOVA followed by Tuckey test).
